# A Case of Pasteurella Bacteremia in a Patient With Hepatocellular Carcinoma

**DOI:** 10.7759/cureus.50161

**Published:** 2023-12-08

**Authors:** Kendrick H Williams, Ashwin Jagadish, Rupal Shah

**Affiliations:** 1 Internal Medicine, East Tennessee State University, James H. Quillen College of Medicine, Johnson City, USA

**Keywords:** blood cultures, internal medicine, bacteremia, pasteurella infection, pasteurella multocida

## Abstract

*Pasteurella multocida *is a Gram-negative coccobacillus commonly associated with soft tissue and skin infections. On rare occasions, it may result in systemic bacteremia and sepsis. Our case describes a 59-year-old male who presented to the emergency department with septic shock. Physical examination was remarkable for bilateral lower extremity wounds which were in recent contact with feline oral secretions. Blood cultures were obtained and resulted in the growth of *P. multocida* after 48 hours. His treatment involved intravenous antibiotics and supportive care. After finishing his two-week course of antibiotics, he was placed on inpatient hospice care due to his clinical course involving other comorbidities and expired shortly after. This case highlights the importance of early recognition and treatment of *P. multocida* infection in patients with comorbid conditions such as hepatocellular carcinoma.

## Introduction

In immunocompetent and non-elderly individuals, skin and soft tissue infections associated with *Pasteurella multocida* typically present as local, uncomplicated infections [1]. *P.* *multocida* is a common member of the normal flora of the oral cavity and upper respiratory tract of both domestic and wild animals, most commonly felines and canines [2]. It is usually transmitted through animal bites or scratches [2]. However, there have been cases reported of *Pasteurella* infection caused by mere contact with oral secretions [2]. One study demonstrated that non-bite *Pasteurella* infections may lead to higher mortality than infections associated with a bite [2].

*P. multocida *evades the immune response through the utilization of a polysaccharide capsule [3]. *Pasteurella* is more prevalent in individuals with declining immune function and reduced capacity to phagocytize encapsulated pathogens [2].

## Case presentation

**Figure 1:**
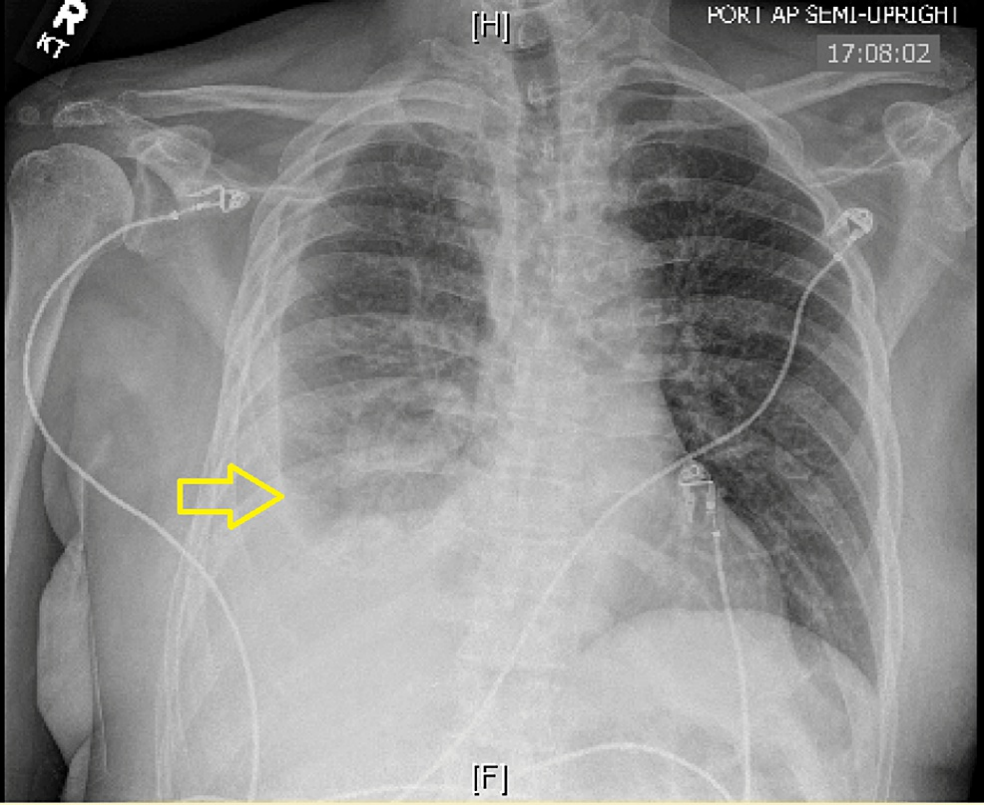
Chest X-Ray The arrow indicates right-sided pleural effusion.

A 59-year-old male with a past medical history of hepatocellular carcinoma and alcoholic cirrhosis presented to the emergency department with hypotension and tachycardia suggesting septic shock. On presentation, his chest X-ray showed a moderate right-sided pleural effusion with consolidation in the right lower lung (Figure 1). Also, his lactate level was 3.9 mmol/L (normal 0.7-2.0 mmol/L). At the time, his only other concern was mild, bilateral lower extremity pain. A comprehensive physical examination revealed multiple bilateral lower extremity wounds. While the patient was uncertain how he obtained the wounds, he reported that the wounds were frequently in contact with his domesticated cat’s oral secretions. Blood cultures tested positive for *P. multocida*. A lower extremity X-ray and further clinical investigation were not indicative of osteomyelitis. The patient was treated with intravenous meropenem and fluid resuscitation. Due to this patient’s other comorbidities, he was placed in inpatient hospice and expired shortly after completing his course of intravenous meropenem.

## Discussion

Our patient presented to the emergency department with fever, hypotension, and tachycardia suggesting septic shock. In addition, he was noted to have an elevated lactate level. A comprehensive history and physical examination revealed that the patient had multiple lower extremity wounds that were in contact with feline oral secretions.

In immunocompetent and non-elderly individuals,* Pasteurella* can present with local skin and soft tissue infections; however, in those who are elderly or immunocompromised, *Pasteurella* infection is more commonly associated with life-threatening complications [4]. These include bacteremia, osteomyelitis, meningitis, and endocarditis [2]. Mortality in individuals with a more complicated presentation can be up to 30% [2]. We hypothesized that our patient’s severe presentation with *Pasteurella *bacteremia is closely associated with his underlying cirrhosis and immunocompromised status attributed to malignancy. Of note, our patient’s Child-Pugh score was calculated as 13. It is also worth mentioning that one study demonstrated that *Pasteurella* bacteremia is more common in patients with cirrhosis and metastatic cancer [5].

Treatment of *P. multocida *bacteremia with signs of systemic infection begins with empiric parenteral antibiotic therapy pending sensitivity reports [1]. Beta-lactam antibiotics are generally effective in treating *P. multocida* [6]. While our patient was initially started on intravenous ceftriaxone, his medication regimen was later changed to intravenous meropenem following sensitivity reports. Consideration of osteomyelitis is important in patients with *Pasteurella *bacteremia. Our patient’s lower extremity X-ray and clinical evaluation suggested that his condition had not progressed to osteomyelitis.

## Conclusions

Generally, invasive complications of *P. multocida *are not present in those who are immunocompetent or non-elderly. However, our case highlights the importance of conducting a thorough history and physical examination in individuals presenting with signs of infection, especially in those who have cirrhosis or malignancy. Additionally, obtaining blood cultures and antibiotic sensitivity reports can help identify the source of infection and guide treatment.

The prognosis of *P. multocida *bacteremia can be variable, depending upon the patient population. Early identification and treatment of disease can help prevent adverse outcomes. Additionally, education and hygiene can also be beneficial.
